# HPLC-CUPRAC post-column derivatization method for the determination of antioxidants: a performance comparison between porous silica and core-shell column packing

**DOI:** 10.1186/s40543-018-0137-1

**Published:** 2018-01-17

**Authors:** Syed A. Haque, Socrates Jose P. Cañete

**Affiliations:** Kentucky Tobacco Research and Development Center, University of Kentucky, 1401 University Drive, Lexington, KY 40546 USA

**Keywords:** Antioxidants, Post-column derivatization, CUPRAC, Core-shell column, Porous silica column

## Abstract

**Background:**

An HPLC method employing a post-column derivatization strategy using the cupric reducing antioxidant capacity reagent (CUPRAC reagent) for the determining antioxidants in plant-based materials leverages the separation capability of regular HPLC approaches while allowing for detection specificity for antioxidants.

**Methods:**

Three different column types, namely core-shell and porous silica including two chemically different core-shell materials (namely phenyl-hexyl and C18), were evaluated to assess potential improvements that could be attained by changing from a porous silica matrix to a core-shell matrix. Tea extracts were used as sample matrices for the evaluation specifically looking at catechin and epigallocatechin gallate (EGCG).

**Results:**

Both the C18 and phenyl-hexyl core-shell columns showed better performance compared to the C18 porous silica one in terms of separation, peak shape, and retention time. Among the two core-shell materials, the phenyl-hexyl column showed better resolving power compared to the C18 column.

**Conclusions:**

The CUPRAC post-column derivatization method can be improved using core-shell columns and suitable for quantifying antioxidants, exemplified by catechin and EGCG, in tea samples.

## Background

The importance of functional nutrition has sparked an active interest in natural antioxidants due to their inherent ability to protect the human body by inhibiting free radical reactions. As a corollary to this, the general public would want to know the constituents and levels of antioxidant capacity in the food we consume. There are numerous published methods claiming to measure total antioxidant capacity (TAC) in vitro; from the list of TAC assays in the literature, the ferric-reducing antioxidant power (FRAP) and cupric reducing antioxidant out (CUPRAC) methods are two most commonly used and are proposed for standardization during the First International Congress on Antioxidant Methods. The reaction mechanism in both the FRAP and CUPRAC methods toward antioxidants is solely electron transfer, and therefore, both methods can provide insight into dominant reaction mechanisms of various antioxidants in a given sample matrix, thus providing a framework for reliable quantification of total antioxidant activity (Prior et al. 2005).

Between the FRAP and CUPRAC methods, the latter has gained significant attention in the past few years because of its advantages in terms of its applicability for both hydrophilic and lipophilic antioxidants, its workability at physiological pH and its faster response (Huang et al. 2005). Since its development by Apak et al., the CUPRAC method for determining antioxidant capacity was employed numerous time by the different groups on various natural products of fruits and vegetables. For instance, TAC levels in multiple samples of apple juice were compared to individual antioxidant levels separately analyzed by HPLC (Karaman et al. 2010). The same approach was also employed to correlate the TAC levels with high-performance liquid chromatography (HPLC) findings for antioxidants present in the leaves of *Petroselinum sativum* (parsley), leaves of *Apium graveolens* (celery), and in *Urtica dioica* (stinging nettle) (Yıldız et al. 2008). In addition, the CUPRAC method for TAC determination has also been employed for the determination of trace level endogenous antioxidants in human serum with proven superior performance than FRAP especially for thiol-containing antioxidants like reduced glutathione (Apak et al. 2010). With its proven performance as a “total assay,” the CUPRAC reagent is an attractive candidate as a derivatization reagent in an HPLC post-column instrumental setup. Conceptually, the separated antioxidant components are allowed to react with the CUPRAC reagent in a post-column mixing unit before reaching the detector, thus leveraging the separation capability of regular HPLC approaches while allowing for detection specificity for antioxidants (Zacharis and Tzanavaras 2013). Multiple variants of this general post-column derivatization approach has been reported in the analysis of antioxidants from the leaves of *Eucommia ulmoides* Oliv. (Dai et al. 2013), from the flower buds of *Lonicer* spp. (Li et al. 2012) and from the leaves of *Sonchus oleraceus* Linn (Ou et al. 2013). All three reports used DPPH as the post-column derivatization reagent. Recently Jones and co-workers reported the use of FRAP reagent for the analysis of antioxidants in coffee (Jones et al. 2017). While proven to be functional, the main issue with HPLC post-column derivatization approaches, in general, is that the more post-column volume the HPLC system has (a necessity for analytes to react with the derivatization reagent), the broader the peaks will be which results in reduced chromatographic resolution.

We report here a method employing a similar post-column approach using CUPRAC as the post-column derivatization reagent. Although the general approach of this technique is not particularly new, improvements could potentially be attained if the column used was changed from a porous silica matrix to a core-shell matrix. The particle morphology of a core-shell column matrix is touted to result in less band broadening compared to fully porous particles and thus presumably delivers extremely high efficiencies and greater resolution (Gritti and Guiochon 2012b). This is a fundamentally logical assumption because in order to maximize efficiency, sources of band broadening need to be minimized. With core-shell particles, sources of band broadening (specifically the A and B terms) as described by the van Deemter equation are reduced compared to fully porous particles. This reduction in band broadening fundamentally results in chromatographic separations with better resolution, higher sensitivity, and improved peak capacities (Gritti and Guiochon 2012a). To our knowledge, this is the first report comparing the porous silica matrix with the core-shell matrix in a post-column derivatization format.

## Methods

### Chemicals

All the chemicals used in the experiments were purchased from either Sigma-Aldrich or TCI America and used as supplied. The HPLC grade solvents were purchased from VWR analytical, USA. The water used for the experiments was purified with a Milli-Q water system (Millipore, USA). Copper (II) chloride dihydrate, neocuproine hemihydrate, and sodium acetate used in the experiments were of the highest purity (> 99%) available. The antioxidants (+) catechin hydrate and (−) epigallocatechin gallate hydrate standards were used as standards for calibration and spike experiments.

### Preparation of CUPRAC reagent

The CUPRAC reagent was prepared by mixing equal volume of 1 mM CuCl_2_*2H_2_O (in methanol/water 50/50), 25 mM Sodium acetate (in acetone/water 25/75), and 2 mM neocuproine (in methanol/water 50/50). The resultant reagent which itself is pale yellow in color, upon reacting with antioxidants, turns bright orange showing a maximum at 450 nm in UV. The reagent was prepared fresh prior to any experiment.

### UV experiments

The UV experiments were done in a UV Microplate Reader (Spectra MAX 140 series, Molecular Devices). Spectra of catechin and EGCG (0.75 mM each in CH3OH/H2O 1:1) were taken with and without presence of CUPRAC reagent. Aliquots of 150 μL of each antioxidant (1.5 mM) were taken into a 96-well UV plate in different time intervals and mixed with 150 μL of CUPRAC reagent, and reading was taken immediately after shaking on an Eppendorf Mixmate shaker for 45 s. A control measurement (for dilution effect) was also performed in which, instead of the antioxidant solution, 150 μL of methanol/water 50/50 was mixed with the CUPRAC reagent and used as plate blank.

### HPLC experiments

The antioxidants were analyzed using an HPLC instrument (Shimadzu LC 2040C) with a post-column mixing pump and a photodiode array (PDA) detector. The chromatographic separation was carried out in a porous silica column (Phenomenex Luna C18, 150 × 4.6 mm, 5 μm, Phenomenex, California, USA) and two core-shell columns (Phenomenex Kinetex C18, 150 × 4.6 mm, 5 μm and Phenomenex Kinetex phenyl-hexyl, 150 × 4.6 mm, 5 μm, Phenomenex, California, USA). Chromatographic separation was carried out using the following gradient system: solvent A, DI water, solvent B, and methanol. Gradient elution profile: 9-min linear gradient to 22% solvent B- 78% solvent A; isocratic elution for 6 min; gradient elution to 30% solvent B- 70% solvent A for another 5 min and return to initial condition via linear gradient for 3-min period. Flow rate of the separation gradient was 1.0 mL/min while that of the derivatization pump was 0.5 mL/min. The PDA detector was set at 450 nm for CUPRAC derivatization. The CUPRAC reagent used as post-column derivatization in the HPLC system is the same as previously described.

### Preparation of tea samples

Tea samples of five commercial brands were procured from a local grocery store; brand 1 is a pure green tea, brand 2 is a blended black tea, brand 3 is a blended green tea, brand 4 is another blend of black tea, and brand 5 is chai black tea. Two grams of each brand were transferred into individual 150 mL Erlenmeyer flasks and extracted with 100 mL of boiled DI water. The mixtures were swirled occasionally, and the extraction was allowed to happen for 20 min. After that time, a 5-mL aliquot of each sample was transferred into a centrifuge tube and centrifuged at 4000 rpm for 10 min. The supernatant layer was syringe filtered using a 0.2-μm PTFE membrane before performing the experiments. Triplicate sample preparations were done on each sample, and two separate HPLC injections were carried out for each replicate preparation.

## Results and discussion

### Detection response–UV experiments

UV-vis spectroscopy was used in order to assess the spectral profiles of the samples and antioxidant standards and to reproduce spectral profiles reported in literature. The CUPRAC reagent is light turquoise with pale yellow overtone and does not show any observable peak between 350 and 650 nm. Upon introduction of an antioxidant, there was an observable change in color to yellow or deep orange in a concentration-dependent manner. It also exhibited concentration-dependent absorbance at 450 nm consistent with reports on CUPRAC assays (Özyürek et al. 2011; Apak et al. 2004). Similarly, when extracts from different tea samples–initially showing no observable peak in the visible region–were introduced into the CUPRAC reagent mixture, they showed peaks at 450 nm consistent with previous reports. Both experiments confirm the reduction of Cu(II)-neocuproine to yellow-colored Cu(I)-neocuproine complex consistent with observations reported in literature. Furthermore, the observed change in color–from turquoise to yellow/yellow orange–was almost instantaneous for both the standards catechin and EGCG as well as the tea extracts. This makes the CUPRAC reagent an attractive and compatible post-column derivatization reagent because the reaction kinetics of post-column derivatization reagents should ideally be fast (Zacharis and Tzanavaras 2013; Apak et al. 2005).

### HPLC experiments: comparison of columns

Peak broadening due to “extra-column effects” has been recognized as one aspect in chromatographic separation that practitioners have thrived to minimize (Fekete and Fekete 2011; Gritti and Guiochon 2010). We have acquiesced to the inevitability of “extra-column effects”–and consequently to potential peak broadening due to extra-column volume––in this post-column derivatization format using CUPRAC, and hence, we looked at other potential avenues at which separation and quantification of antioxidants using this format can be improved. In this regard, columns packed with core-shell particles can be a good candidate.

**Fig. 1:**
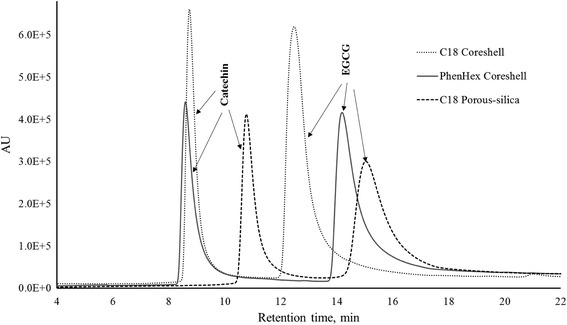
Comparative chromatograms of standard antioxidants with CUPRAC post-column derivatization reagent using porous silica and core-shell column stationary phases

After checking the UV spectra and determining workable wavelength, the samples were subjected to HPLC experiments using porous silica and core-shell columns for separation, identification, and quantification. Identification of catechin and EGCG was done using standards of the two antioxidants. Performances of the columns were evaluated by running a mixture of standard catechin and EGCG; the comparative chromatograms are shown in Fig. 1. At the outset, the two peaks for all three columns were nicely resolved. The inherent characteristic common to these peaks–observable in all three columns–is that they are relatively broad which is somewhat expected considering that there is a significant degree of extra-column volume; a characteristic that is unavoidable in post-column setups (Zacharis and Tzanavaras 2013). Evaluating the resolution and full width-half maximum values of these peaks show that the use of core-shell stationary phase gives better resolved peaks (where resolution, R = 2*(*t*_*R*, EGCG_ *– t*_*R*, catechin_)/(*W*_EGCG_ *+ W*_catechin_)) and narrower band width compared to using porous silica. Furthermore, since band broadening is directly related to plate height (*H*) as theoretically described in the van Deemter equation, and plate height is inversely proportional to the number of theoretical plates, *N* (where *N = L*_column_/*H*), we have compared the plate heights between the two core-shell columns and a porous silica column (Table 1). For both antioxidants, the number of theoretical plates (*N*) for the core-shell columns is higher than the porous silica which strongly suggests that the use of core-shell particles in the column could enhance column performance in the analysis of antioxidants in this post-column derivatization format.

**Table 1:** Peak and separation characteristics for catechin and EGCG standards

Antioxidant	Column material	Full width at half maximum (min)	Base width (*W*)	Retention time (*t*)	No. of theoretical plates (*N*)
Catechin	C18 porous silica	0.470 ± 0.015	3.824	10.77 ± 0.01	2912 ± 170
	C18 core shell	0.351 ± 0.057	3.688	8.73 ± 0.04	3431 ± 616
	Phenyl-hexyl core shell	0.358 ± 0.054	4.936	8.59 ± 0.02	3193 ± 675
EGCG	C18 porous silica	1.127 ± 0.052	9.440	15.02 ± 0.08	985 ± 71
	C18 core shell	0.712 ± 0.121	6.232	12.47 ± 0.03	1701 ± 867
	Phenyl-hexyl core shell	0.774 ± 0.089	8.168	14.19 ± 0.01	1864 ± 480

Computed resolution values: *R*_C18 porous silica_ = 0.641; *R*_C18 core shell_ = 0.754; *R*_Phen-hex core shell_ = 0.855

Although our present investigation is not exhaustive, a wealth of experimental data show that the measured column performance is strongly affected by extra-column band broadening especially pronounced in columns packed with particles smaller than the standard 4.6 μm (Fountain et al. 2009). Literature reporting sub-2 μm particle column characterization identified “extra-column effects” as a factor negative correlated with column performance (Heinisch et al. 2008; Usher et al. 2008; Cabooter et al. 2008).

Early reports on the benefits of using solid core materials in chromatographic columns have been associated with reduced transfer effects, the “C” term in the van Deemter equation. However, more recent reports indicate that these benefits were mostly contributed by dispersion processes, the “A” and “B” terms. The notion that solid core support materials provide a benefit to chromatographic performance by virtue of the A term (Eddy diffusion) is well supported with experimental data (Hayes et al. 2014). This is a very logical notion considering that the A term is directly related to particle size, structure, and packing efficiency of support particle of the column and solid core materials, pack better than fully porous materials. The solid core material provides a rougher surface and a better packing efficiency (Tanaka and McCalley 2016; Hayes et al. 2014). In our experiments, the particle sizes for both the porous silica and core shell are the same (5 μm) for the both column materials.

Furthermore, a significant reduction in dead volume is also expected in solid core materials compared to fully porous materials; a characteristic that relates to better column performance that can be theoretically explained with the B term. Gritti and co-workers reported that a fully porous material packed in a column will occupy only one-third of the column volume while the solid core material increases the occupied volume by 20–30% (Gritti et al. 2011). It is also recognized that the minimal value of longitudinal diffusion as reflected in the B term is obtained in part when the particles have negligible porosity or when the analyte is poorly retained or not retained at all. The limit of column improvement by virtue of the B term when using solid core materials is below the optimal linear velocity, i.e., low flow rates, as improvements are theoretically negligible at, or above, this optimal linear velocity (Hayes et al. 2014). The longitudinal diffusion (the B term) which contributes the most on band broadening at low velocity is associated with two parameters: the change in diffusion of the analyte in the presence of the stationary phase particles and the particle’s diffusion coefficient in the mobile phase. Gritti and co-workers simplified the B term based on the Garnett-Torquato model where it was shown that longitudinal diffusion can be minimized by increasing the ratio of solid core diameter to the whole diameter of the particle as it reduces the dead volume of the column (Gritti and Guiochon 2011; Gritti and Guiochon 2014). Although much of the benefits of the solid core derived from the A and B terms, the C term or resistance to mass transfer comes into effect for the bigger molecules and negligible for small ones (Gritti and Guiochon 2005; Gritti et al. 2006). Our experiments, by design, used EGCG (molar mass 458) being much bigger than catechin (molar mass 290) such that mass transfer effects could potentially be observed when separated in both fully porous and core-shell columns. However, these effects were not observed in our experiments. We presume that compared to the sizes of proteins and peptides which were intensively studied by Gritti and Guiochon, the sizes of the antioxidants used here may be too small to exhibit such mass transfer effects typically observed in proteins and peptides.

### HPLC experiments: calibration and linearity

Given the differences in performance of the fully porous and core-shell columns, we proceeded to perform quantitative calibration to determine the linearity of concentration-dependent response and method detection limits for purposes of quantifying catechin and EGCG in tea samples. Calibration curves for the antioxidants catechin and EGCG were generated from fresh solutions and used for the quantification where each point in the calibration curve is the average of triplicate injection. The peak areas corresponding to each antioxidants were calculated automatically by the HPLC software (LabSolutions), and a linear relationship was established by plotting the average peak areas against concentration.

**Fig. 2:**
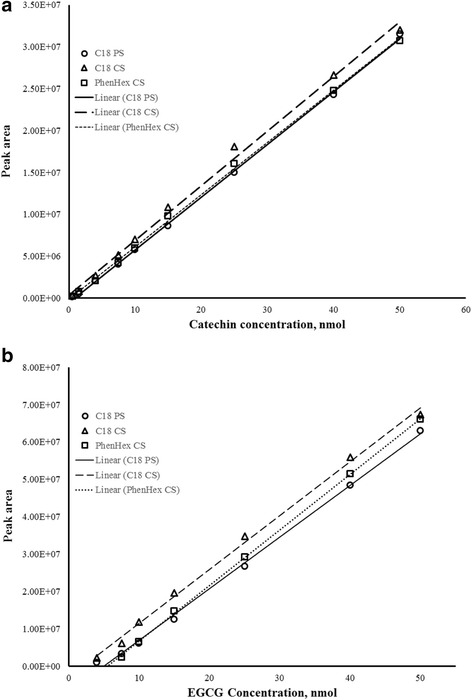
HPLC calibration curves for antioxidants catechin (**a**) and EGCG (**b**) using C18 porous silica (solid lines), C18 core-shell (coarse dashed lines) and phenyl-hexyl core-shell (fine dashed lines) columns with CUPRAC post-column derivatization

Calibration curves using the C18 porous silica, C18 core-shell and phenyl-hexyl core-shell columns showed comparably good linearity relative to both catechin and EGCG peaks (Fig. 2). In addition, all three columns exhibited comparable sensitivities. The limit of detection (LOD) for the method using the three columns was also approximated by multiple blank readings. The calibration linear regression, detection sensitivity, and limits of detection using the three columns are summarized in Table 2. Given that the detection sensitivity of the method is comparable using the three different columns, it would be expected that the estimated LOD using these columns would also be comparable. This is typified by the analysis of EGCG. For catechin, on the other hand, the method LOD using the C18 porous silica column appears to be slightly higher relative to both the core-shell columns. This could be due to signal noise in the expected peak area of catechin (i.e., from expected start to expected end of peak) where the C18 porous silica column has more variable blank readings at which the standard deviation of the blanks is higher for C18 porous silica column relative to the two core-shell columns.

**Table 2:** Linear regression, sensitivity, and limits of detection using different columns

Antioxidant	Column material	Regression (*r*^*2*^)	Sensitivity, slope	LOD (nmol)
Catechin	C18 porous silica	0.922	6.30E + 05	0.56
	C18 core shell	0.996	6.53E + 05	0.38
	Phenyl-hexyl core shell	0.999	6.23E + 05	0.39
EGCG	C18 porous silica	0.999	1.41E + 06	0.24
	C18 core shell	0.997	1.42E + 06	0.29
	Phenyl-hexyl core shell	0.999	1.49E + 06	0.29

At this point, it appears that the linear regression, sensitivity, and detection limits are comparable for all three columns when using this post-derivatization format. However, the fact that the peaks come out earlier for the core-shell columns with increased resolution relative to the porous silica column—despite the inevitable peak broadening due to “extra-column effects”—these characteristics of the core-shell columns could be leveraged to achieve higher analysis throughput without sacrificing sensitivity.

### Quantification of antioxidants in tea

**Fig. 3:**
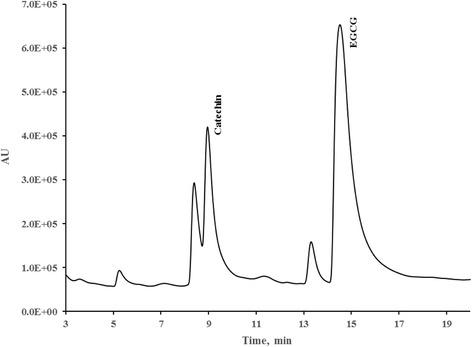
Spiking experiment and identification of catechin and EGCG in tea extract matrix using phenyl-hexyl core-shell column

Since the linearity and approximated detection limits observed and computed for all three columns were adequate for quantification antioxidants in the part-per-million range, the method was used to evaluate the levels of catechin and EGCG in tea samples. Introduction of tea extracts into the HPLC system coupled with PDA detector yielded observable separation of antioxidants including catechin and EGCG. We looked at only these two antioxidants in our studies because these are two of the four most common flavanols in tea. Furthermore, their chemical structures are very distinct with projected retention times far apart that these two antioxidants can better serve to illustrate the difference in performance between the core-shell column and the porous silica column. Identity of each antioxidant in the tea extract matrix was confirmed by a set of spiking experiment. Spiking experiments were also conducted for proper identification of peaks within the tea extract matrix as well as the basis for the measurement of trueness (Fig. 3). In our experiments, the recovery was between 80 and 90% for all three columns. For a concentrations in the part-per-million (ppm) range, a recovery between 80 and 110% is normally acceptable without correction; therefore, there was no quantitative correction applied to the final quantification of catechin and EGCG in the tea samples analyzed.

**Fig. 4:**
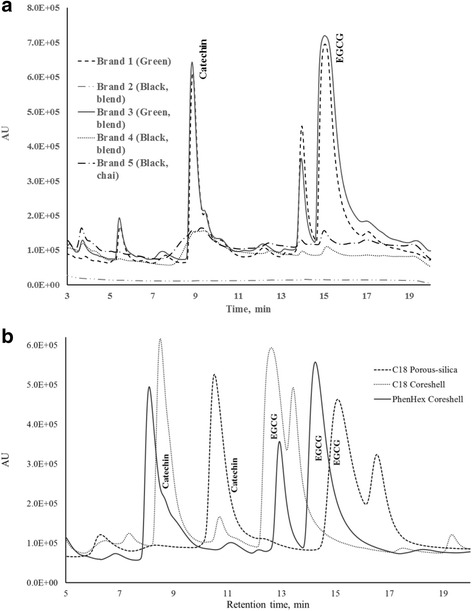
**a** HPLC chromatograms of extracts for five tea samples of different type and brand with CUPRAC post-column derivatization using phenyl hexyl core-shell column. **b** Comparative chromatograms of a tea sample (brand 1 green tea extract) with CUPRAC post-column derivatization reagent using C18 porous silica, C18 core-shell, and phenyl-hexyl core-shell columns

Among the two core-shell columns with different stationary phases, there were noticeable differences in their separation profiles. It was observed that using the same gradient conditions, both the C18 porous silica and the C18 core shell could not resolve the overlapping peaks of catechin and another antioxidant species–most likely epicatechin–while the phenyl-hexyl core-shell column was able to partially resolve these two peaks (Fig. 4). This disparity in stationary phase performance could be due to the unique selectivity of the phenyl-hexyl stationary phase toward aromatic compounds. The observed unresolved overlap in the two C18 columns could potentially result in sub-par quantification for catechin as the gradient system used was not optimal for these columns; for these reasons, quantification was not done on data from the C18 porous silica and C18 core-shell columns. Furthermore, the absence of peak overlap in the phenyl-hexyl column demonstrates that the better separation between catechin and epicatechin is not due to the change in physical property (from porous silica to core shell); rather, it is because of the chemical constituent of the column. With triplicate preparation of each sample (*n* = 3) and two separate injections per replicate sample preparation, the quantification of two antioxidants in the tea samples done using phenyl-hexyl core-shell column is presented in Table 3. Among the five tea samples, two green teas have significantly higher amount of flavan-3-ol antioxidants particularly catechin and EGCG. EGCG is the most abundant antioxidant comprising around 50 mg per gram of green tea sample. Black teas are lacking flavan-3-ols probably due to the extensive fermentation processing for black teas which significantly reduces the levels of these antioxidants in the final product.

**Table 3:** Antioxidants present per gram of tea samples (extracted in hot water for 20 min) separated in an HPLC with CUPRAC post-column derivatization using phenyl-hexyl core-shell column

Antioxidants	Brand 1 (*n* = 3)	Brand 2 (*n* = 3)	Brand 3 (*n* = 3)	Brand 4 (*n* = 3)	Brand 5 (*n* = 3)
Catechin, mg/g	12.16 ± 0.24 (RSD = 1.97%)	0.68 ± 0.06 (RSD = 8.82%)	12.94 ± 0.17 (RSD = 1.31%)	0.45 ± 0.02 (RSD = 4.44%)	0.69 ± 0.05 (RSD = 7.25%)
EGCG, mg/g	40.71 ± 2.76 (RSD = 6.77%)	1.30 ± 0.01 (RSD = 0.78%)	61.57 ± 0.44 (RSD = 0.72%)	0.62 ± 0.16 (RSD = 25.81%)	1.09 ± 0.09 (RSD = 9.17%)

## Conclusions

We have evaluated the performance of an HPLC method for the analysis of antioxidants with enhanced detection via post-column derivatization using CUPRAC employing three columns–two types of support matrices (porous silica and the core shell) and two types of stationary phases with the same support matrix (core shell, C18, and phenyl-hexyl stationary phases). Broad peaks were observed for all three columns which are a consequence of extra-column volume, but there was significantly less broadening in the core-shell column data. Theoretical considerations support the notion that the core-shell column would perform better than the porous silica column by virtue of the A and B terms in the van Deemter equation while the C term is negligible due to the small molecular sizes of the antioxidants. The C18 core-shell column exhibited sharper peaks with higher plate efficiency compared to the C18 porous silica column with shorter retention times. Among the two core-shell columns, the phenyl-hexyl column had better resolving power for the aromatic analytes which is predictable for its phenyl group. The separation and analysis of antioxidants in real samples–as demonstrated here using five brands of tea–can also benefit from the use of core-shell columns as it provides better and quicker separation (i.e., shorter retention times) compared to porous silica. While the linear regression, detection sensitivity, and detection limits are comparable for all three columns in this CUPRAC post-column derivatization approach, the characteristically shorter analyte retention times observed for the core-shell columns could be advantageous in achieving higher analysis throughput without sacrificing analytical sensitivity.
